# Ovine *ENSOARG00020011332* rs427117280-GG provides an increase in weaning weight and average daily gain until weaning weight in a multi-breed sheep population

**DOI:** 10.5194/aab-68-357-2025

**Published:** 2025-05-28

**Authors:** Bilal Akyüz, Fadime Daldaban, Mustafa Özdemir, Ferda Karakuş, Davut Bayram, Serdar Yağcı, Ali Atik, Şeyma Kökkaya, Korhan Arslan, Emre Şirin, Mehmet Ulaş Çınar

**Affiliations:** 1 Department of Genetics, Faculty of Veterinary Medicine, Erciyes University, 38039 Kayseri, Türkiye; 2 Department of Animal Science, Faculty of Agriculture, Erciyes University, 38039 Kayseri, Türkiye; 3 Department of Animal Science, Faculty of Agriculture, Van Yüzüncü Yıl University, 65080 Van, Türkiye; 4 Department of Animal Science, Faculty of Veterinary Medicine, Erciyes University, 38039 Kayseri, Türkiye; 5 General Directorate of Agricultural Research and Policies, Ministry of Agriculture and Forestry, 06800 Ankara, Türkiye; 6 Department of Animal Researches, Bahri Dagdas International Agricultural Research Institute, 42020 Konya, Türkiye; 7 Department of Veterinary Genetics and Biotechnology, Institute of Health Sciences, Erciyes University, 38039 Kayseri, Türkiye; 8 Department of Agricultural Biotechnology, Faculty of Agriculture, Kırşehir Ahi Evran University, 40100 Kırşehir, Türkiye; 9 College of Veterinary Medicine, Department of Veterinary Microbiology and Pathology, Washington State University, Pullman, WA 99164, USA

## Abstract

Lamb meat, which is consumed as widely as pork, beef and poultry, has a very important place in red-meat consumption worldwide. Sheep can survive without consuming green fodder in geographies with poor pastures and very harsh continental climate conditions. In lambs reared for meat purposes, the average daily weight gain (ADG) from birth to weaning and their live weight at weaning are crucial economic traits that affect the profitability of breeders. Additionally, lamb live weight at weaning age could be vital for the lamb's subsequent live weight gain and survival rate. Therefore, improving the live weight at weaning age is desired to increase lamb meat production. However, information on animals' genetic background that affects the weaning weight is limited in many sheep breeds. The aim of this study was to investigate the association of the rs427117280 single nucleotide polymorphism (SNP) on chromosome 2 (OAR2) with live weight gain at different time points (live weight at birth and on the 30th, 60th and 90th days post-birth) in indigenous sheep breeds (Akkaraman, Şavak Akkaraman, Morkaraman, Karayaka and İvesi) in Türkiye. In total, 1973 lambs were genotyped using Custom TaqMan SNP genotyping assays for the rs427117280 SNP. Association analysis showed that lambs with a GG genotype had the highest weight on the 90th day (BW90) and the highest ADG from birth to the 90th day (ADGB-90) ( P≤0.01 ). It was concluded that rs427117280 could be considered as a candidate quantitative trait nucleotide (QTN) for the BW90 and ADGB-90 traits. Therefore, these findings suggested that variation in the rs427117280 SNP might be used as novel genetic marker for improving pre-weaning and weaning growth traits in sheep.

## 1 Introduction

Sheep were domesticated from the Asian mouflon (*Ovis orientalis*) approximately 11 000 years ago (BP) in the Fertile Crescent, the northern border of which is formed by the Southeastern Anatolia region of Türkiye (Zeder, 2008). The domestic sheep (*Ovis aries*), a very important agricultural species in socioeconomic terms in developing and underdeveloped countries, plays a vital role in the livelihood of a significant portion of the population engaged in animal husbandry (Saadatabadi et al., 2021). Global sheep breeds, including Türkiye's 33 different native sheep breeds and their hybrids, exhibit significant genetic and phenotypic variation in terms of growth traits (Aydin et al., 2024; Gürsoy, 2006; Yilmaz et al., 2014; Öner et al., 2014; Kizilaslan et al., 2022; Kar et al., 2024).

The Food and Agriculture Organization of the United Nations (FAO) anticipates that the global human population will reach 9.15 billion individuals by 2050. This increase in population is anticipated to result in a higher demand for animal products in the future (Fitzmaurice et al., 2020). Meeting this raised demand for livestock products and avoiding negative environmental impacts will require the more efficient utilization of natural resources (Michalk et al., 2019). Sheep farming has a crucial environmental role worldwide with respect to reducing both the impact of greenhouse gas emissions and the carbon footprint of farming practices, as the carbon footprint of sheep meat production is reported to be lower than that of beef production (Mazzetto et al., 2023; Lal et al., 2022). Additionally, sheep are also valuable livestock for societies that meet their red-meat needs from ruminants. Another advantage of sheep compared to cattle is that they are more profitable. Fixed and variable costs are the same for both species; however, sheep have a substantially higher live weight increase value than cattle (USD 4.79 vs. USD 1.46 per kg) (Piltz et al., 2021). Lamb live weight and live weight gain have been defined as the key drivers of profitability in international sheep production systems (Cocks et al., 2002; Conington et al., 2004). Byrne et al. (2010) reported that each additional day that a lamb requires to attain its desired slaughter weight results in an economic loss of EUR 1.41 per lamb per day for Irish sheep farmers. Thus, obtaining lambs that reach their slaughter weight quickly is essential for profitable sheep breeding. In sheep, growth and reproduction traits are closely linked, as both traits are influenced by genetic and environmental factors, and improvements in one area can often impact the other (Safari et al., 2005). A positive correlation has been reported between the live weight of the offspring at weaning and their subsequent growth and reproductive characteristics in sheep (Pickering et al., 2012; Mohammadi et al., 2014), suggesting that selection for growth traits can also enhance reproductive performance (Ramos et al., 2023).

Genome-wide association studies (GWASs) using known single nucleotide polymorphisms (SNPs) in farm animals have revealed genetic variants as potential genetic markers for desirable traits in order to maintain genetic gain (Akhatayeva et al., 2020). In our previous GWAS, a quantitative trait nucleotide (QTN; rs427117280; OAR2: 248863817G > T) was detected (genome-wide) to be significantly associated with weaning weight and daily weight gain until weaning in Akkaraman lambs (Cinar et al., 2023). The validation of GWAS results in multi-breed livestock is crucial to enhance the accuracy of genomic selection and understand genetic traits across different breeds (van den Berg and MacLeod, 2023). Therefore, the objective of this study was to investigate the associations of the rs427117280 SNP with lamb live weight and lamb live weight gain until weaning at different time points in five indigenous sheep (Akkaraman, Şavak Akkaraman, Morkaraman, Karayaka and İvesi) breeds in Türkiye.

## 2 Material and methods

### 2.1 Ethics statement

This study was conducted according to the experimental protocols approved by the Animal Care and Use Committee of Erciyes University (no. 23/016-01.02.2023).

### 2.2 Experimental animals and DNA isolation

A total of 1973 lambs of five different breeds (Akkaraman, n=467 ; Şavak Akkaraman, n=361 ; Morkaraman, n=453 ; Karayaka, n=316 ; and İvesi, n=376 ) from 17 herds were utilized to document body weights (BWs) until weaning in the Kayseri, Ağrı, Tokat, Mersin and Erzincan provinces of Türkiye. Animals subjected to this study were bred according to “National Sheep and Goat Breeding Program and Breeder Associations' Collaboration Systems”, managed by the Ministry of Agriculture and Forestry in Türkiye. In the context of the program, birth and weaning weights were recorded regularly to establish elite sheep flocks (https://www.tarimorman.gov.tr, last access: 25 October 2024). Commercial husbandry techniques were implemented during the experiment. The flocks were pastured except during December and March, which coincided with the peak lambing period. During housing, animals were provided a diet consisting of wheat stubble, barley and alfalfa. The breeding season occurred from August to October, during which time ewes were randomly allocated to rams in natural settings. Lambing commenced in late December and persisted into March. The date of birth was documented for all lambs, and they were promptly ear-tagged post-lambing. Creep feed (ad libitum) was provided to lambs starting 15 d prior, and they were weaned at approximately 90 ± 2.0 d of age. The lambs were weighed at birth and on the 30th (BW30), 60th (BW60) and 90th (BW90) days post-birth to determine weaning weight. The average daily gain (ADGB-90) was calculated by dividing the weight gained by a lamb since the last measurement by the number of days elapsed since that measurement. Following the measurement of each lamb's weight on the 90th day, 10 mL of blood was collected from the jugular veins into the tubes containing ethylenediaminetetraacetic acid. DNA was extracted from blood samples utilizing a commercial kit in accordance with the manufacturer's guidelines (Qiagen, Hilden, Germany). A NanoDrop ND-1000 spectrophotometer (NanoDrop Technologies, Wilmington, DE, USA) was employed to assess the quantity and quality of the isolated DNA. DNA samples were adjusted to a final concentration of 50 $\mathrm{ng}\;µL^{-1}$ and preserved at -20 °C until required.

### 2.3 SNP genotyping using the TaqMan method

Using Custom TaqMan SNP genotyping assays (Thermo Fisher Scientific., Waltham, MA, USA), 1973 lambs were genotyped for the rs427117280 SNP (OAR chromosome 2: 248863817G > T). Allelic discrimination primers and hydrolysis probes (Thermo Fisher Scientific, Waltham, MA, USA) were used to genotype rs427117280 (forward: ACGTTCTTTAAGCAC ACCCGTTTA; reverse: GTTGTAGTCCGCATTGAGAAACG, VIC-ACCTTGCTGCACCGCA and FAM-AACCTTGCTTCACCGCA). The final reaction volume was 10 µL , and the remaining components were 50 ng of DNA and TaqMan PCR Genotyping Master Mix (Applied Biosystems, Foster City, CA, USA). A total of 5 µL of TaqMan Genotyping Master Mix, 0.5 µL of TaqMan probe, 2 µL of DNA and 2.5 µL of water made up the PCR reaction mixture. The C1000 Touch Thermal Cycler (Bio-Rad, CA, USA) was used to carry out the PCR; we ran 40 cycles at 95 °C for 15 s, 40 cycles at 60 °C for 1 min and 1 cycle at 95 °C for 10 min. The ABI Prism StepOnePlus Real-Time PCR System (Applied Biosystems, Foster City, CA, USA) was used to image the genotypes following the PCR process.

### 2.4 Statistical analyses

Lamb data were checked for normality before analyses with the “sasLM” package (version 3.4.1) in R (version 4.4.1). The Hardy–Weinberg equilibrium (HWE) and the allele and genotype frequencies of SNPs in this study were calculated using “SNPassoc” (version 2.0–11) in R (version 4.4.1). The genotype–phenotype association was examined utilizing a generalized linear model with the “GLM” function of the “sasLM” package (version 3.4.1) in R (version 4.4.1). The simplified model used fixed effects for genotype (3 levels), sex (2 levels), breed nested within farms (17 levels), birth (2 levels) and sheep age. Genotypic comparisons were presented after Tukey–Kramer correction, with a significance threshold set at P≤0.01 .

1 $$y_{imcjl}=\mu +G_{i}+O_{m}+F_{c}+T_{j}+N_{l}+e_{imcjl}$$

In the expression above, $y_{imcjl}$ is phenotype (birth weight and the weights and daily live weight gain on the 30th, 60th and 90th days), μ is the population mean, $G_{i}$ is the fixed effect for genotype (3 levels: GG, TG or TT), $O_{m}$ is sex (2 levels: male or female), $F_{c}$ is the farm impact (17 levels), $T_{j}$ is the fixed effect for birth type (2 levels: single or twin) and $N_{l}$ is the fixed effect for ewe age.

## 3 Results

The rs427117280 SNP showed deviation from the HWE in all breeds except Karayaka, and the distributions of the genotypes and allele frequencies of lambs are shown in Table 1. It was observed that the heterozygous TG genotype was the most common in the examined sheep (Table 1).

**Table 1:** The rs427117280 genotype and allele frequencies.

SNP	Breeds ( n )	Genotypes frequencies			Allele		HWE ( $\chi^{2}$ )
					frequencies		
		TT	TG	GG	T	G	
rs427117280	All animals (1973)	260 (0.13)	1457 (0.74)	256 (0.13)	0.5	0.5	448.81^∗^
	Akkaraman (467)	42 (0.089)	407 (0.872)	18 (0.039)	0.53	0.47	260.12^∗^
	Şavak Akkaraman (361)	30 (0.083)	255 (0.706)	76 (0.211)	0.44	0.56	68.64^∗^
	Morkaraman (453)	32 (0.071)	363 (0.801)	58 (0.128)	0.46	0.54	154.45^∗^
	Karayaka (316)	127 (0.402)	142 (0.449)	47 (0.149)	0.63	0.37	0.5^NS^
	İvesi (376)	29 (0.077)	290 (0.771)	57 (0.152)	0.46	0.54	114.22^∗^

In the right-hand column, $\chi^{2}$ represents chi-square, ^∗^ denotes P<0.001 and NS represents not significant.

The rs427117280 SNP was found to be associated with the 90th-day weight and average daily gain from birth to the 90th day (ADGB-90) in the examined lambs (Table 2). It was observed that lambs with the GG genotype had a statistically higher ( P≤0.01 ) 90th-day live weight and daily live weight gain from birth to the 90th day compared to lambs with other genotypes (Table 2). In the breed-wise analysis, the Şavak Akkaraman breed showed an association with the BW90 and ADGB-90 ( $P=2.037\times 10^{-10}$ and $P=1.051\times 10^{-10}$ , respectively) (Table S2 in the Supplement). Although no other breed (in addition to the Şavak Akkaraman animals) was found to be statistically associated with the BW90 and ADGB-90 (Tables S2), the rs427117280-GG genotype tended to have a high BW90 and ADGB-90 in the Akkaraman, Morkaraman, Karayaka and İvesi breeds (Tables S1, S3, S4 and S5 in the Supplement).

**Table 2:** Association of *ENSOARG00020011332* rs427117280 with lamb live weights and lamb live weight gain until weaning, showing the adjusted means and standard errors, for the multi-breed sheep population.

SNP	Genotypes	Birth weight (g)	BW30 (g)	BW60 (g)	BW90 (g)	ADGB-90 (g)
rs427117280	GG	3746.60±23.0	11471.20±197.0	17973.20±278.0	25367.77±346.0	240.23±3.8
	TG	3771.80±12.0	11108.10±109.0	17594.87±155.0	24339.82±193.0	228.53±2.1
	TT	3726.60±24.0	11044.10±206.0	17508.00±292.0	24168.57±363.0	227.12±4.01
	Birth type	^∗∗∗^	^∗∗^	^∗∗^	^∗∗∗^	^∗^
	Breed (farm)	^∗∗∗^	^∗∗∗^	^∗∗∗^	^∗∗∗^	^∗∗∗^
	Sex	^∗∗∗^	^∗∗∗^	^∗∗∗^	^∗∗∗^	^∗∗∗^
	Ewe age	^∗∗^	^∗∗^	^∗^	NS	NS
	$R^{2}$	0.39	0.45	0.38	0.44	0.45
	P value	0.17	0.18	0.39	**0.01**	**0.01**

The abbreviations used in the table are as follows: BW – body weight; ADGB-90 – average daily weight gain until 90th day. Bold lettering highlights genotypes that are significantly associated with traits of interest ( P≤0.01 ). Significant levels are denoted as follows: ^∗∗∗^ represents P≤0.001 , ^∗∗^ represents P≤0.01 , ^∗^ represents P≤0.05 and NS represents not significant.

## 4 Discussion

Many significant economic traits in livestock, including growth and daily live weight gain, adhere to a polygenic complicated inheritance model. Consequently, research that concentrates on candidate genes that are responsible for the emergence of polygenic inherited traits in livestock has always been of interest in order to support genetics and breeding (Goddard and Hayes, 2009). As in all livestock, growth traits in sheep breeding directly affect production costs and profitability. Therefore, studies to improve sheep breeding in terms of live weight traits can be achieved more quickly through genetic selection (Mrode et al., 2018). The results of the research done towards this aim have led to the suggestion that certain genes and SNPs can be utilized to enhance the traits associated with livestock's overall live weight gain (Goddard and Hayes, 2009; Armstrong et al., 2018). Compared to other farm animals, there are often less studies on the identification of genes and SNPs linked to growth and meat yield traits in sheep (Zhi-Liang et al., 2022). Against the background of the limited application of genetic study results, the search for SNPs that can be used as potential markers for live weight gain is crucial, especially with respect to increasing the yield of low-yielding local sheep breeds. For this purpose, in the current study, the relationships between the birth weight and the 30th-day, 60th-day, and 90th-day (weaning age) weight or average daily gain until weaning age and the rs427117280 SNP were investigated in five local sheep breeds (Akkaraman, Şavak Akkaraman, Morkaraman, Karayaka and İvesi) in Türkiye. The rs427117280 SNP has not previously been studied in any other sheep breed except Akkaraman (Cinar et al., 2023); therefore, the findings of the current research can shed new light on breeding for live weight and live weight gain in lambs.

In the study conducted by Cinar et al. (2023), it was reported that the rs427117280 SNP in OAR2 is close to the natriuretic peptide precursor-C (*NPPC*) gene, a potential candidate gene associated with growth. C-type natriuretic peptide (CNP), a member of the natriuretic peptide family that plays an important role in regulating endochondral bone development, shows its effect by binding to a cell surface receptor, natriuretic peptide receptor 2 (NPR2) (Çetin et al., 2022; Hisado-Oliva et al., 2018). In mammals, the expression level of the natriuretic peptide precursor-C (*NPPC*) gene encoding CNP changes according to the growth (infancy or adult) period of the animal. While this gene is primarily expressed in the uterus, ovaries, forebrain and brainstem in adult mice, it is also expressed in different tissues and organs, such as skeletal muscle, liver and kidney, in newborns (Stepan et al., 2000). *NPPC* plays a critical role in growth in mammals, as it is expressed in many tissues, although mostly in the hypertrophic zone of the growth plate (Olney, 2006; Peng et al., 2013; Vasques et al., 2014). On the other hand, it has been reported that CPN knockout mice have impaired growth and develop dwarfism, while transgenic mice overexpressing CNP grow excessively (Olney, 2006; Hisado-Oliva et al., 2018). Therefore, *NPPC* appears to be a potential candidate gene for growth. However, different types of studies need to be done to confirm this.

While it was seen in the literature review that there were studies reporting *NPPC* as a potential candidate gene associated with body weight gain in farm animals (Kijas et al., 2012; Fariello et al., 2014; Xu et al., 2015; Rochus et al., 2018; Edea et al., 2020; Li et al., 2020; Cinar et al., 2023), it was also noted that there was only one study investigating the relationship between mutations or polymorphisms in this gene and growth and live weight gain. The study, conducted by Cinar et al. (2023) in 373 native Akkaraman lambs, showed the relationship between the rs427117280 SNP and live weight at weaning age. It was reported that the examined samples were in HWE for the SNP and that TT was the most common genotype (0.911), whereas GG was the least common genotype (0.001) in the investigated population (Cinar et al., 2023). However, the current study, in which 1973 lambs were genotyped from five local Turkish sheep breeds, exhibited deviation from HWE for the rs427117280 SNP, and the frequencies of the TT and GG genotypes were found to be equal to each other (0.13). While the lambs studied by Cinar et al. (2023) were the offspring of parents raised using extensive sheep breeding methods, the lambs in the current study were the offspring of parents selected according to the herd book, in which phenotypes (body weights at birth and weaning) were recorded systematically. This may be the cause of deviation from HWE in the investigated population.

In a two-stage study conducted by Cinar et al. (2023), they first reported that the rs427117280 SNP was associated with both the BW90 (weaning age) and ADGB-90 in 192 Akkaraman lambs that they examined as a result of a GWAS. In the second stage of their study, Cinar et al. (2023) genotyped 373 Akkaraman lambs for the rs427117280 SNP and showed that this SNP was associated with the BW90 and ADGB-90. In the present study, in which 1973 lambs from fat-tailed (Akkaraman, Şavak Akkaraman, Morkaraman and İvesi) and thin-tailed (Karayaka) sheep breeds raised in Türkiye were examined, it was revealed that the rs427117280 SNP was associated with both the BW90 and ADGB-90. In both the study conducted by Cinar et al. (2023) and our study, it was observed that lambs with the rs427117280-GG genotype were superior to those with other genotypes in terms of the BW90 and ADGB-90.

There is a high correlation between weaning weight and post-weaning weight (0.75) and adult weight (0.93) (Medrado et al., 2021; Safari et al., 2005; Snyman et al., 1998). Breeding studies focusing on the birth weight and weaning weight are important to increase meat yield in farm animals. Among other agricultural species, sheep are versatile farm animals raised for meat, milk and wool production. A high correlation (0.78–0.80) has been reported between weaning weight and fertility in sheep (Medrado et al., 2021; Zishiri et al., 2013). Therefore, selection studies focused on improving the live weight at weaning in sheep will not only increase meat yield but may also contribute to fertility traits and wool yield. Thus, both the current study and the study by Cinar et al. (2023) showed that the rs427117280 SNP can be used in selection studies for the BW90 and may also provide more profitability for post-weaning traits such as body weight and fertility in sheep.

## 5 Conclusion

Taking these result together, the rs427117280 SNP can be utilized as a molecular marker affecting the weaning and average daily weight gain until weaning in various sheep breeds. It was observed that the rs427117280-GG genotype frequency was still very low in the five different sheep breeds examined. Therefore, it is thought that selection studies to increase the frequency of this genotype in sheep breeds will make a positive contribution to increasing the live weight at weaning. Similarly, more extensive research may be designed to validate the association of rs427117280 with pre-weaning and weaning growth traits in different sheep breeds globally, and protein and mRNA expression studies for liver, bone and skeletal muscle tissues can be designed to elucidate more information regarding understanding the molecular mechanism of the *NPPC* gene in sheep growth traits. We believe that studies examining larger numbers of samples from different sheep breeds should be conducted to reveal the effects of this SNP on the examined traits.

## Supplement

10.5194/aab-68-357-2025-supplement The supplement related to this article is available online at https://doi.org/10.5194/aab-68-357-2025-supplement.

## Data Availability

The datasets generated are available from the corresponding author upon request.
