# Supplementary material for: Ovine ENSOARG00020011332 rs427117280-GG provides an increase in weaning weight and average daily gain until weaning weight in a multi-breed sheep population
Source: Arch Anim Breed. 2025 May 28;68(2):357–63. doi: 10.5194/aab-68-357-2025 (PMC13283029; doi:10.5194/aab-68-357-2025)
Supplement: The supplement related to this article is available online at https://doi.org/10.5194/aab-68-357-2025-supplement. [file aab-68-357-2025-supplement.pdf]

Supplement of Arch. Anim. Breed., 68, 357–363, 2025
https://doi.org/10.5194/aab-68-357-2025-supplement
© Author(s) 2025. CC BY 4.0 License.

Open Access
Archives Animal Breeding

Supplement of

Ovine *ENSOARG00020011332* rs427117280-GG provides an increase in weaning weight and average daily gain until weaning weight in a multi-breed sheep population

Bilal Akyüz et al.

Correspondence to: Mehmet Ulaş Çınar (mucinar@erciyes.edu.tr)

The copyright of individual parts of the supplement might differ from the article licence.

Table S1. Association of *ENSOARG00020011332 rs427117280* with growth traits weights showing adjusted means and standard errors from Akkaraman sheep population.

SNP	Genotypes	Birth Weight	BW30	BW60	BW90	ADGB-90
rs427117280	GG	3,781.02±26.0	14,077.40 ±603.0	18,703.05 ±331.0	30,899.77±346.0	301.43±4.8
	TG	3,839.73±12.0	13,441.94 ±127.0	18,816.73 ±150.0	30150.82±193.0	292.68±2.1
	TT	3,792.80±26.0	13,473.17 ±396.0	17,508.00±336.0	30914.57±363.0	301.73±4.8
	Birth type	***	***	**	**	***
	Farm	NA	NA	NA	NA	NA
	Sex	NS	***	***	***	***
	Ewe age	*	*	NS	NS	NS
	R^2	0.31	0.10	0.11	0.15	0.15
	P-value	0.20	0.58	0.55	0.46	0.41

*BW: Body weight; ADGB-90: Average daily weight gain until 90th day; Bold lettering highlights when genotypes were significantly associated with traits of interest ($P \leq 0.01$); *** ($P \leq 0.001$); ** ($P \leq 0.01$); * ($P \leq 0.05$); NS: not significant; NA: not applicable.*

Table S2. Association of *ENSOARG00020011332 rs427117280* with growth traits showing adjusted means and standard errors from Şavak Akkaraman sheep population.

SNP	Genotypes	Birth Weight	BW30	BW60	BW90	ADGB-90
rs427117280	GG	38,56.27±23.0	8,298.85±228.0	14,609.87 ±278.0	20,676.71 ±346.0	186.89±3.8
	TG	39,08.24±12.0	7,359.94±151.0	13,506.83 ±155.0	17,417.55 ±193.0	150.10±2.1
	TT	38,59.59±24.0	7,534.90±359.0	12,832.35 ±292.0	16,735.74 ±363.0	143.06±4.01
	Birth type	***	NS	NS	NS	NS
	Farm	***	***	*	**	***
	Sex	***	NS	NS	NS	*
	Ewe age	NS	NS	NS	NS	NS
	R^2	0.35	0.10	0.038	0.13	0.14
	P-value	0.56	**0.0006**	**0.004**	**2.037e-10**	**1.051e-10**

*BW: Body weight; ADGB-90: Average daily weight gain until 90th day; Bold lettering highlights when genotypes were significantly associated with traits of interest ($P \leq 0.01$); *** ($P \leq 0.001$); ** ($P \leq 0.01$); * ($P \leq 0.05$); NS: not significant; NA: not applicable.*

Table S3. Association of *ENSOARG00020011332 rs427117280* with growth traits showing adjusted means and standard errors from Karayaka sheep population.

SNP	Genotypes	Birth Weight	BW30	BW60	BW90	ADGB-90
rs427117280	GG	3,713.85±23.0	13,215.29 ±228.0	20,201.12 ±278.0	28,284.26 ±346.0	273.00±3.8
	TG	3,694.44±12.0	12,078.55 ±151.0	18,596.30 ±155.0	26,577.30 ±193.0	254.25±2.1
	TT	3,675.70±24.0	12,005.54 ±359.0	18,982.69 ±292.0	26,569.86 ±363.0	254.37±4.01
	Birth type	**	NS	NS	NS	NS
	Farm	***	NS	***	***	***
	Sex	*	NS	*	***	***
	Ewe age	***	NS	NS	NS	NS
	R^2	0.58	0.005	0.10	0.19	0.17
	P-value	0.82	0.25	0.26	0.30	0.30

*BW: Body weight; ADGB-90: Average daily weight gain until 90th day; Bold lettering highlights when genotypes were significantly associated with traits of interest (P ≤ 0.01); *** (P ≤ 0.001); ** (P ≤ 0.01); * (P ≤ 0.05); NS: not significant; NA: not applicable.*

Table S4. Association of *ENSOARG00020011332 rs427117280* with growth traits showing adjusted means and standard errors from Morkaraman sheep population.

SNP	Genotypes	Birth Weight	BW30	BW60	BW90	ADGB-90
rs427117280	GG	3,781.64±23.0	9,154.80±228.0	15,487.23 ±278.0	28,284.26 ±346.0	208.77±3.8
	TG	3,796.89±12.0	9,651.66±151.0	16,386.82 ±155.0	26,577.30 ±193.0	223.11±2.1
	TT	3,760.58±24.0	9,301.72±359.0	15,744.15 ±292.0	26,569.86 ±363.0	218.84±4.01
	Birth type	***	***	***	***	***
	Farm	NS	NS	NS	NS	NS
	Sex	NS	NS	NS	NS	NS
	Ewe age	NS	NS	**	**	***
	R^2	0.29	0.13	0.16	0.14	0.12
	P-value	0.79	0.30	0.15	0.12	0.21

*BW: Body weight; ADGB-90: Average daily weight gain until 90th day; Bold lettering highlights when genotypes were significantly associated with traits of interest (P ≤ 0.01); *** (P ≤ 0.001); ** (P ≤ 0.01); * (P ≤ 0.05); NS: not significant; NA: not applicable.*

Table S5. Association of *ENSOARG00020011332 rs427117280* with growth traits showing adjusted means and standard errors from İvesi sheep population.

SNP	Genotypes	Birth Weight	BW30	BW60	BW90	ADGB-90
rs427117280	GG	3,685.50±23.0	14,918.22 ±228.0	20,298.50 ±278.0	28,639.98 ±346.0	277.27±3.8
	TG	3,748.50±12.0	14,928.72 ±151.0	20,475.58 ±155.0	28,373.22 ±193.0	273.60±2.1
	TT	3,611.79±24.0	14,928.72 ±359.0	20,381.72 ±292.0	27,110.45 ±363.0	261.09±4.01
	Birth type	***	**	**	**	*
	Farm	***	***	***	***	***
	Sex	***	***	***	***	***
	Ewe age	NS	***	***	***	***
	R^2	0.45	0.49	0.48	0.52	0.52
	P-value	0.18	0.99	0.94	0.34	0.39

*BW: Body weight; ADGB-90: Average daily weight gain until 90th day; Bold lettering highlights when genotypes were significantly associated with traits of interest ($P \leq 0.01$); *** ($P \leq 0.001$); ** ($P \leq 0.01$); * ($P \leq 0.05$); NS: not significant; NA: not applicable.*
